# The effect of sequencing errors on metagenomic gene prediction

**DOI:** 10.1186/1471-2164-10-520

**Published:** 2009-11-12

**Authors:** Katharina J Hoff

**Affiliations:** 1 Department of Bioinformatics, Institute of Microbiology and Genetics, Georg-August-University Göttingen, Göttingen, Germany; 2 International Max Planck Research School for Molecular Biology, Georg-August-University Göttingen, Göttingen, Germany

## Abstract

**Background:**

Gene prediction is an essential step in the annotation of metagenomic sequencing reads. Since most metagenomic reads cannot be assembled into long contigs, specialized statistical gene prediction tools have been developed for short and anonymous DNA fragments, e.g. MetaGeneAnnotator and Orphelia. While conventional gene prediction methods have been subject to a benchmark study on real sequencing reads with typical errors, such a comparison has not been conducted for specialized tools, yet. Their gene prediction accuracy was mostly measured on error free DNA fragments.

**Results:**

In this study, Sanger and pyrosequencing reads were simulated on the basis of models that take all types of sequencing errors into account. All metagenomic gene prediction tools showed decreasing accuracy with increasing sequencing error rates. Performance results on an established metagenomic benchmark dataset are also reported. In addition, we demonstrate that ESTScan, a tool for sequencing error compensation in eukaryotic expressed sequence tags, outperforms some metagenomic gene prediction tools on reads with high error rates although it was not designed for the task at hand.

**Conclusion:**

This study fills an important gap in metagenomic gene prediction research. Specialized methods are evaluated and compared with respect to sequencing error robustness. Results indicate that the integration of error-compensating methods into metagenomic gene prediction tools would be beneficial to improve metagenome annotation quality.

## Background

Metagenomes are analyzed through simultaneous sequencing of all species in a microbial community without prior cultivation under laboratory conditions. The result is usually a large collection of sequencing reads from many species, and the phylogenetic origin of each read is unknown. A major goal in all metagenomic studies is the identification of potential protein functions and metabolic pathways. Reliable gene predictions are the basis for correct functional annotation, and for the discovery of new genes with their functions.

Several gene prediction methods have been developed for the *ab initio *identification of protein coding genes in complete microbial genomes (e.g. GLIMMER and GeneMark [1,2]). These methods require an initial training phase on some data from the target genome, or training on the genome of a closely related species. Such *conventional *gene finders can in principle be applied to metagenomic data, given that single sequencing reads can be *assembled *into longer contigs in order to provide sufficient training data. The applicability of *conventional *gene finders to metagenomic contigs can be improved by *binning *contigs and reads into separate phylogenetic scaffolds, e.g. by their oligonucleotide signature [3]. However, the assembly of metagenomic sequencing reads is problematic. Mavromatis *et al*. (2007) demonstrated on artificial metagenomes that assembly quality highly depends on the sequencing coverage of single species within the metagenome [4]. They also showed that short contigs are at high risk of chimerism, i.e. a read from species A is joined with a read of species B, which limits the use of contigs for further analysis. Some proportion of most metagenomes remains in single unassigned sequencing reads after assembly and binning, and in some cases, metagenome assembly fails completely, e.g. for the hypersaline microbial mat metagenome [5]. For this reason, the ability of predicting genes in single and anonymous sequencing reads is essential to fully explore a metagenome.

This problem can be solved by two strategies. One possibility is the identification of protein coding regions through sequence similarity. An example is to conduct a BLAST search [6] with metagenomic sequences against a database of known proteins. Annotation success is here limited to already known genes and their close relatives. This problem is particularly prominent for viral sequences that are poorly represented in databases [7-9]. Clustering of open reading frames (ORFs) in principle enables sequence similarity based methods to identify novel genes that are conserved within the metagenomic sample [10,11]. Considering the size of most metagenomes, computational cost is a limiting factor for these methods.

A different strategy is based on gene prediction with statistical models. GeneMark with heuristic models [12], MetaGene [13], Orphelia [14,15] and MetaGeneAnnotator [16] fall into the category of model-based metagenomic gene prediction tools. The common advantage of these tools is the capability to predict known and novel genes at a lower computational cost. Their mostly unexplored disadvantage is the susceptibility to sequencing errors - which methods that are based on sequence similarity may automatically compensate to a certain extent.

The possible effect of sequencing errors on model-based metagenomic gene prediction depends on the actual error rates. The two major sequencing techniques that are commonly used in metagenomics have different sequencing accuracy. Chain termination sequencing [17] was the first method to be used for metagenome sequencing. It produces an average read length of ~700 nucleotides (nt). The error rates reported for Sanger sequencing vary from 0.001% [13,18] to more than 1% [19,20] and seem to depend on the software that is used for post processing of reads. Pyrosequencing, also known as "454 sequencing", produces shorter reads [21,22]. In the beginning, read length was about 110 nt and has now increased to more than ~450 nt. Huse *et al*. (2007) reported an error rate of 0.49% for reads of the length 100-200 nt [23], the read simulation software MetaSim [20] produces reads with an error rate of 2.8% with parameters that are adjusted according to an original 454 publication [22]. Pyrosequencing is still subject to constant research. In the near future, a further increase in read length can be expected.

For all techniques, sequencing accuracy is high at the beginning of a read and decreases with read length. Three error types can occur: (1) substitution errors, that means a wrong nucleotide is read out, (2) deletion errors, in which one or more nucleotides are omitted, and (3) insertion errors, where one or more nucleotides are falsely added to the sequence during the reading process.

All statistical gene prediction tools utilize codon usage as an important feature to identify protein coding genes. If a nucleotide is deleted or inserted into the sequence, this causes a shift in the reading frame. Methods that do not compensate for frame shifts cannot predict affected genes accurately. Substitution errors will only affect one codon and their influence on gene prediction accuracy is therefore generally smaller. All types of errors may also result in additional stop codons. False stop codons may have an even more severe effect on gene prediction than a frame shift because they will definitely terminate a predicted gene.

The robustness with respect to sequencing errors in Sanger reads has been investigated and discussed for MetaGene and Orphelia [13,14], other tools have not been evaluated with regard to this property. In particular, no studies about the effect of sequencing errors in 454 reads on metagenomic gene prediction are available. Three benchmark data sets that were supposed to facilitate the accuracy evaluation of metagenome analysis tools on real data were introduced [4] but so far, metagenomic gene prediction tools have not been evaluated on these data sets.

In this work, we demonstrate the extent to which typical errors in Sanger and pyrosequencing reads affect metagenomic gene prediction. The effect strongly depends on the actual error rate. For investigation, we utilize sequences simulated with MetaSim, a metagenome simulator [20]. Gene prediction quality on the metagenomic benchmark data sets is also shown and discussed. ESTScan [24], a tool for the curation of expressed sequence tags (ESTs), was trained for the application to metagenomes, and gene prediction accuracy results of ESTscan lead us to the conclusion that the integration of error compensating methods into metagenomic gene prediction tools might significantly improve their performance, and with this metagenome annotation quality.

## Results

### Simulated reads

The evaluation of metagenomic gene prediction tools is complicated by a lack of reliably annotated metagenomic reads. The annotation quality of complete genomes can be expected to be much better. For this reason, we used simulated reads from annotated genomes for the evaluation of metagenomic gene prediction tools.

The models underlying all metagenomic gene prediction tools were built on the basis of genomes from selected training species. Generalization capabilities of those models can only be analyzed if training species and their close relatives (we define *close relative *as *species from the same genus*) are excluded from the evaluation setup. In this study, we did not aim at the simulation of realistic microbial communities, but instead, we wanted to encompass a wide range of phyla. Therefore, a set of prokaryotic microorganisms was selected according to this criterion (see Table 1). None of these species has a genus relative in the training data of metagenomic gene predictions tools investigated in this study.

**Table 1:** Test species - species whose genomes were used to simulate sequencing reads.

Species	Phylum	GC-content
*Acholeplasma laidlawii PG-8A*	Termicutes	31%
*Buchnera aphidicola str. APS*	Proteobacteria (*β*)	30%
*Burkholderia pseudomallei K96243*	Proteobacteria (*γ*)	68%
*Chlorobium tepidum TLS*	Bacteriodetes/Chlorobi group	56%
*Corynebacterium jeikeium K411*	Actinobacteria	61%
*Desulfurococcus kamchatkensis 1221n*	Crenarcheota	45%
*Dictyoglomus thermophilum H-6-12*	Dictyoglomi	33%
*Exiguobacterium sibiricum 255-15*	Firmicutes	47%
*Herpetosiphon aurantiacus ATCC 23779*	Chloroflexi	50%
*Hydrogenobaculum sp. Y04AAS1*	Aquificae	34%
*Natronomonas pharaonis DSM 2160*	Euryarchaeota	63%
*Nitrosopumilus maritimus SCM1*	Crenarcheota	34%
*Prochlorococcus marinus str. MIT 9312*	Cyanobacteria	31%
*Wolbachia endosymbiont strain TRS of Brugia malayi*	Proteobacteria (*α*)	34%

Sanger sequencing reads with the average length of 700 nt were simulated with error rates ranging from 0 to 1.5%, and 454 reads that are on average 450 nt long were simulated with error rates ranging from 0 to 2.8%. All simulated reads are available at http://metagenomic-benchmark.gobics.de.

### ESTScan matrix

ESTScan is a tool that was originally developed to detect coding regions in eukaryotic ESTs and simultaneously correct sequencing errors [24]. In contrast to metagenomic gene prediction methods, ESTScan cannot detect overlapping coding regions as they frequently occur in prokaryotic genomes. We were interested in ESTScan's sequencing error correction capabilities. In order to test if ESTScan would also compensate errors in coding regions on metagenomic sequencing reads, we trained an ESTScan scoring matrix on prokaryotic genomes that were also used for training MetaGene and Orphelia. The matrix is availabe at http://metagenomic-benchmark.gobics.de.

### Accuracy on unassembled simulated reads

Gene prediction accuracy can be estimated by measuring the overlap of predicted and annotated genes in the same reading frame. A comparison on amino acid sequence level reflects overlap and reading frame if the basic requirement that the sequences are highly similar (almost identical) is fulfilled. An amino acid sequence alignment with only few missmatches and gaps shows this kind of similarity. We used BLAT [25] alignments of predicted and annotated genes to assess gene prediction accuracy. Three classes of genes were defined: (1) predicted genes that had a BLAT alignment of at least 20 amino acids (aa) length and at least 80% sequence identity were called true positives, (2) annotated genes that did not fall into the first category were counted as false negatives, and (3) predicted genes that did not have a match with the annotation according to the first criterion were counted as false positives. With these counts, we measured the proportion of annotated genes that were predicted (sensitivity) and the proportion of predicted genes that match genes in the annotation (specificity). Further details are given in section Methods.

**Figure 1:**
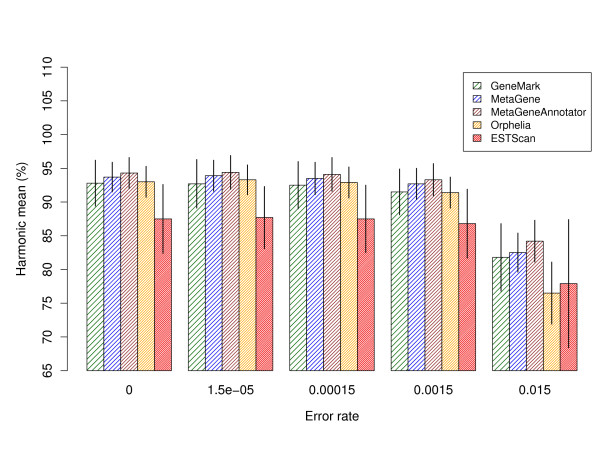
**Average gene prediction accuracy on simulated Sanger reads**. The harmonic mean is a measure that combines sensitivity and specificity (mean and standard deviation over all species in the simulated metagenome are shown).

On simulated Sanger sequencing reads, MetaGene and MetaGeneAnnotator show the highest gene prediction sensitivities (~94% over all species on error free reads and ~80% on reads with the highest error rate) while Orphelia has the best specificity values with ~96% on error free reads and ~92% on reads with the highest error rate (compare Table 2). This result is in agreement with previous publications [14,15]. To estimate overall gene prediction accuracy, sensitivity and specificity were combined in a harmonic mean. Results are visualized in Figure 1. Generally, the MetaGeneAnnotator shows the highest accuracy. The accuracy of all tools decreases only very mildly (by ~1.4%) on reads from 0.0015% to 0.15% errors. Also common to all tools is a drastic drop in accuracy of ~10% from reads with 0.15% errors to reads with 1.5%. For a concise picture, we also measured amino acid prediction accuracy. For this, all amino acids that were captured into a BLAT alignment of prediction and annotation were counted as true positives. All amino acids in the annotation that were not predicted by the true positive criterion were counted as false negatives, and the remaining predicted amino acids were counted as false positives.

**Table 2:** Accuracy on simulated Sanger reads.

	GeneMark		MetaGene		MGA		Orphelia		ESTScan	
Error rate^1^	Sens.^2^	Spec.^3^	Sens.^2^	Spec.^3^	Sens.^2^	Spec.^3^	Sens.^2^	Spec.^3^	Sens.^2^	Spec.^3^
0 to 0	91.9 ± 3.2	93.8 ± 4.9	94.4 ± 3.0	93.0 ± 2.9	94.7 ± 2.9	94.1 ± 2.9	89.7 ± 3.5	96.5 ± 1.7	78.9 ± 7.2	98.5 ± 1.2
1 to 2 × 10^-5^	91.9 ± 3.3	93.7 ± 5.2	94.8 ± 2.8	93.0 ± 3.0	94.8 ± 2.9	94.0 ± 3.1	90.1 ± 3.3	96.7 ± 1.6	79.2 ± 6.5	98.6 ± 1.1
1 to 2 × 10^-4^	91.8 ± 3.3	93.5 ± 5.2	94.5 ± 2.9	92.6 ± 3.2	94.5 ± 3.0	93.7 ± 3.1	89.6 ± 3.5	96.5 ± 1.7	79.0 ± 7.0	98.4 ± 1.3
1 to 2 × 10^-3^	90.5 ± 3.2	92.6 ± 4.8	93.3 ± 2.8	92.1 ± 3.0	93.3 ± 3.0	93.3 ± 2.8	87.2 ± 3.6	96.0 ± 1.7	78.0 ± 7.2	98.2 ± 1.2
1 to 2 × 10^-2^	77.7 ± 4.4	86.6 ± 6.9	79.8 ± 3.6	85.6 ± 3.9	81.2 ± 4.3	87.6 ± 3.1	65.7 ± 6.4	91.9 ± 1.8	66.2 ± 11.1	96.2 ± 1.8

The gene prediction accuracy (mean and standard deviation over all species in the simulated metagenome) results of four metagenomic gene prediction tools GeneMark, MetaGene, MetaGeneAnnotator (MGA) and Orphelia, and of the EST processing tool ESTScan on simulated Sanger reads is shown.

All tools have an amino acid sensitivity of ~95 to 96% on error free reads. Orphelia and MetaGeneAnnotator have with 97% the highest specificity [see Additional file 1, Table S1]. These values are higher than the corresponding gene prediction accuracy, indicating that long genes on error free reads are more likely to be predicted correctly than short genes. On reads with high error rates (0.15% and 1.5%), we observed that gene prediction rates were higher than amino acid prediction rates. The reason is that long open reading frames are likely to be affected by sequencing errors in form of in frame stop codons. Consequently, only shorter genes can be predicted, which is also confirmed by length boxplots of genes predicted in reads with different error rates [see Additional file 1, Figure S1].

ESTScan had with ~87% a lower harmonic mean (gene prediction level) than metagenomic gene prediction tools on reads with few errors (see Figure 1). Interestingly, the decrease in performance on reads with 0.015% errors to 0.15% errors was only 0.7%, which is smaller than the decrease observed for metagenomic gene prediction tools (ranging from 0.8 to 1.4%). From reads with 0.15% to 1.5% errors, a decrease in accuracy of 9% was observed, which is also a smaller accuracy drop than measured for metagenomic gene prediction tools.

**Figure 2:**
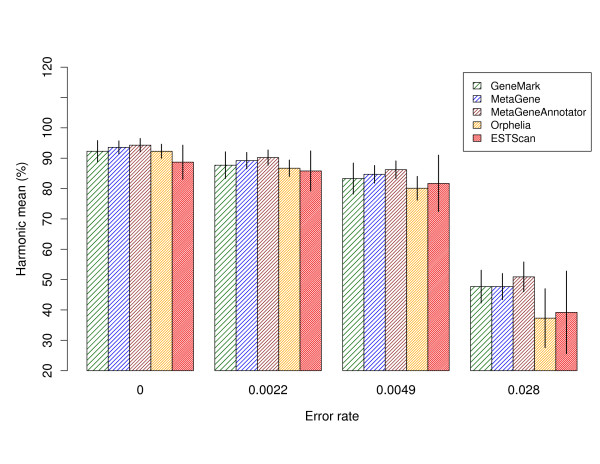
**Average gene prediction accuracy on simulated 454 reads**. The harmonic mean is a measure that combines sensitivity and specificity (mean and standard deviation over all species in the simulated metagenome are shown).

On error free 450 nt pyrosequencing reads, gene prediction sensitivity and specificity of all tools is similar to accuracy on Sanger reads (see Table 3). Also here, MetaGeneAnnotator has the highest gene prediction harmonic mean (94%) as depicted in Figure 2. From error free reads to reads with 0.49% errors, a drop in harmonic mean of ~9% is observed for all gene prediction methods (except for Orphelia with 12%). Opposed to this, ESTScan again showed a smaller decrease of 7%. Continuing to reads with an error rate of 2.8%, a further accuracy decrease of ~35 (MetaGeneAnnotator, MetaGene, GeneMark) to ~42% (Orphelia and ESTScan) follows. On amino acid level, we observe the same effects as for Sanger reads [see Additional file 1, Table S2].

**Table 3:** Accuracy on simulated 454 reads.

	GeneMark		MetaGene		MGA		Orphelia		ESTScan	
Error rate	Sens.^2^	Spec.^3^	Sens.^2^	Spec.^3^	Sens.^2^	Spec.^3^	Sens.^2^	Spec.^3^	Sens.^2^	Spec.^3^
0	91.0 ± 3.6	93.8 ± 4.8	95.4 ± 2.8	92.8 ± 2.4	94.6 ± 2.7	94.1 ± 2.5	88.4 ± 3.5	96.7 ± 1.7	81.3 ± 7.8	97.9 ± 1.4
0.0022	85.3 ± 4.2	90.4 ± 5.6	89.3 ± 3.1	89.2 ± 3.5	89.6 ± 3.3	90.8 ± 2.6	80.0 ± 4.2	94.7 ± 2.1	77.2 ± 9.0	97.2 ± 1.5
0.0049	79.5 ± 4.9	87.6 ± 6.4	83.7 ± 3.5	85.9 ± 3.9	84.7 ± 4.0	87.7 ± 2.8	70.9 ± 5.9	92.5 ± 2.1	71.7 ± 11.5	96.2 ± 1.7
0.028	36.8 ± 4.9	68.3 ± 8.0	39.6 ± 3.9	60.6 ± 8.8	43.3 ± 5.5	61.9 ± 3.6	26.3 ± 9.1	68.3 ± 5.0	26.4 ± 11.2	86.2 ± 4.7

The gene prediction accuracy (mean and standard deviation over all species in the simulated metagenome) of four metagenomic gene prediction tools GeneMark, MetaGene, MetaGeneAnnotator (MGA) and Orphelia, and of the EST processing tool ESTScan on simulated 454 reads is shown.

On the example of 454 reads with an error rate of 0.49%, we further investigated to which extent the GC-content of a read influences gene prediction accuracy. (GC-content is the percentage of bases cytosine and guanine in all bases of a sequence.) Table 4 shows that GeneMark has a higher gene prediction accuracy on low-GC species than on species with a high GC-content. For MetaGene and MetaGeneAnnotator, this effect is smaller, and for Orphelia and ESTScan, we can not find an obvious difference. On the same dataset, we also measured gene prediction sensitivity and specificity for different gene lengths. For measuring sensitivity, we evaluated predicted genes with all lengths against annotated genes of certain length categories (up to 40 aa length, 41 to 80, 81 to 120, 121 to 160, 161 to 200).

**Table 4:** Accuracy by Species.

	GeneMark		MetaGene		MGA		Orphelia		ESTScan	
Species	Sens.^2^	Spec.^3^	Sens.^2^	Spec.^3^	Sens.^2^	Spec.^3^	Sens.^2^	Spec.^3^	Sens.^2^	Spec.^3^
GC-content 30 - 39%										

*B. aphidicola*	81.7	92.6	88.5	88.1	90.1	88.6	66.0	93.5	71.4	98.3
*A. laidlawii*	86.2	94.6	90.4	89.9	91.9	91.3	75.3	96.2	81.4	97.3
*P. marinus*	81.8	91.3	84.0	87.1	84.2	87.9	61.9	92.5	69.3	97.3
*D. thermophilum*	85.1	94.2	84.7	90.5	86.6	91.2	71.8	94.6	70.5	98.5
*N. maritimus*	85.7	90.4	86.2	86.7	87.7	87.7	68.1	92.4	76.9	96.1
*W. endosymbiont*	80.1	81.0	83.6	84.0	84.7	85.6	65.2	90.9	67.6	94.5
*Hydrogenobaculum sp.*	83.9	93.9	84.3	89.9	85.5	90.8	67.2	93.1	73.0	98.1

GC-content 40 - 49%										

*D. kamchatkensis*	73.3	94.3	77.1	89.5	79.5	90.4	61.4	93.1	35.0	94.4
*E. sibiricum*	78.9	90.0	81.7	88.2	81.8	89.4	78.3	93.9	74.4	95.1

GC-content 50 - 59%										

*H. aurantiacus*	70.2	80.1	80.2	84.9	78.6	86.1	74.0	92.7	76.4	95.5
*C. tepidum*	74.0	76.6	78.6	81.0	79.0	83.0	72.7	89.9	72.7	93.7

GC-content 60 - 69%										

*C. jeikeium*	77.2	83.7	84.2	83.3	86.6	88.2	79.7	93.2	76.1	96.3
*N. pharaonis*	77.0	83.5	83.3	81.6	83.9	84.8	72.8	91.3	75.3	94.4
*B. pseudomallei*	77.7	80.5	85.2	77.6	85.9	83.3	77.0	87.8	84.5	97.7

The gene prediction accuracy (mean and standard deviation over all species in the simulated metagenome) of four metagenomic gene prediction tools GeneMark, MetaGene, MetaGeneAnnotator (MGA) and Orphelia, and of the EST processing tool ESTScan on pyrosequencing reads (450 nt) with 0.49% errors.

Specificity was measured by evaluating predicted genes of the length categories against annotated genes of all lengths. All tools most accurately predict genes of 160 aa length or longer (those genes mostly span the complete read). MetaGene and MetaGeneAnnotator have the highest sensitivity on shorter genes while Orphelia has the highest specificity among gene prediction tools on shorter genes [see Additional file 1, Figure S2].

### Accuracy on FAMeS reads

The "Fidelity of Analysis of Metagenomic Samples" (FAMeS) benchmark data sets consist of sequencing reads from single species genome projects [4]. The benchmark data sets were designed to measure the accuracy of assemblers, binning methods and gene prediction methods. Particularly for assessing assembly and binning accuracy, the reads were combined into three sets with different degrees of representation for each species. The low-complexity data set (simLC) consists of reads from mostly one species with a few reads from less abundant species. The medium-complexity data set (simMC) resembles a moderately complex community with more than one dominant population and also has few reads from less abundant species. The high-complexity data set (simHC) lacks dominant populations. However, detecting a difference in gene prediction accuracy between the three different sets will only show that a tool is better for predicting genes in one species than in others. We used the unassembled reads of all FAMeS data sets to test gene prediction accuracy of tools that were mainly designed for the application to single reads or short contigs. Regarding the results, one must consider that the sequence quality of FAMeS raw reads is rather low because the reads are untrimmed, meaning that their low-quality ends have not been removed. On all three data sets, GeneMark shows the highest harmonic mean (~81%) [see Additional file 1, Figure S3]. The most sensitive method on all data sets was MetaGene (78% on simMC and simHC, 80% on simLC), and the highest specificities were observed for GeneMark (86% on simLC, 85% on simMC, 83% on simHC, see Table 5).

**Table 5:** Accuracy on FAMeS reads.

	simLC^4^		simMC^5^		simHC^6^	
Method	Sens.^2^	Spec.^3^	Sens.^2^	Spec.^3^	Sens.^2^	Spec.^3^
GeneMark	78.8	85.9	77.3	85.1	77.1	83.0
MetaGene	80.0	78.4	78.8	77.5	78.0	74.9
MetaGeneAnnotator	79.6	80.2	78.4	79.4	77.3	75.6
Orphelia	76.7	85.0	74.9	82.5	74.8	82.0
ESTScan	70.2	96.0	69.3	96.1	69.0	95.0

The gene prediction accuracy of four metagenomic gene prediction tools GeneMark, MetaGene, MetaGeneAnnotator (MGA) and Orphelia, and of the EST processing tool ESTScan on FAMeS reads [4] is shown.

Interestingly, ESTScan performs almost as good as GeneMark on FAMeS reads (most likely due to the low sequence quality of the FAMeS data set).

## Discussion

The major question of this study was how sequencing errors affect metagenomic gene prediction accuracy. On Sanger reads, prediction accuracy is only mildly affected by error rates of up to 0.15%. Sequencing accuracy in this range is realistic for most combinations of chain termination sequencers and read postprocessing software. In general, all tools are therefore applicable to metagenomic Sanger sequences. The FAMeS data set gives an example for low quality Sanger sequences. On those reads, the accuracy of specialized metagenomic gene prediction tools is not much higher than the performance of conventional gene prediction tools (see [4]). The quality of these reads is very likely to be improved by read postprocessing steps, e.g. by trimming read ends. The results on the FAMeS data set demonstrate that such postprocessing steps are important to ensure a high gene prediction accuracy.

In 2008, Wommack *et al*. showed that a read length of 400 nt significantly weakens BLAST analysis results from metagenomic data [9]. For gene prediction tools that use statistical models, our findings on simulated 450 nt pyrosequencing reads suggest that the actual read length of 450 nt has almost no impact on gene prediction accuracy results when compared to accuracy on 700 nt Sanger reads (compare Tables 2 and 3). Sequencing errors lead to a decrease in gene prediction accuracy, though. On reads with a realistic error rate of 0.49% for pyrosequencing, the harmonic mean is around 82%, which leaves much room for improvement. The identification of short gene fragments, particularly gene fragments shorter than 120 aa (360 nt), is a major shortcoming of metagenomic gene prediction tools. Therefore, gene prediction in pyrosequencing reads would benefit from further development of models specialized on the accurate detection of short gene fragments.

Another interesting question is whether metagenomic gene prediction tools that were trained on a limited set of species genomes are able to predict genes in reads from distantly related species, and whether it is possible to name a 'best tool' for this purpose. The simulated reads used in this study were sampled from species that belong to many different phyla. Members of some phyla were used for training of all tools, other phyla were not represented by a training species. We show that gene prediction accuracy varies over reads from different test species but we believe that this variation is independent from the degree of relatedness to training species. *Dictyoglomus thermophilum *is an example whose phylum was excluded from training of all tools. No significant drop in accuracy can be observed for reads from this species (see Table 4).

On the simulated reads here, it looks like MetaGeneAnnotator is the best tool. In contrast to this, GeneMark has the highest accuracy on FAMeS reads - which are constituted from different species than the simulated data set. Also the results of Hoff *et al*. (2009) demonstrated a high prediction accuracy for GeneMark on a different simulated data set [15]. From the presented data, it is not possible to conclude whether metagenomic gene prediction tools work better or worse for reads from particular phyla because most phyla are represented by only one species. However, it seems that gene prediction accuracy of single tools depends on the species contained in a metagenome.

The accuracy of metagenomic gene prediction tools on real sequencing reads affects further steps of metagenome analysis that generally depend on the predictions. For example, the functional annotation of genes is often achieved by using HMMER [26] or fast tools like UFO [27] for sensitive protein domain database search, e.g. to detect Pfam domains (e.g. [28,29]). The profile hidden Markov models for HMMER or the UFO algorithm cannot detect domains correctly if the predicted genes that are used for database search are affected by frame shift errors within the domain region. In addition, gene prediction sensitivity directly influences the number domains that can potentially be detected. A high gene prediction accuracy ensures that such gene prediction dependent steps of analysis can also be carried out with high accuracy. We showed that ESTScan, although not designed for metagenomic gene prediction, is in principle capable of compensating for frame shift errors in metagenomic data to some extent. Therefore, metagenomic gene prediction accuracy could be greatly improved by the integration of methods that are robust with respect to sequencing errors.

## Conclusion

In conclusion, the integration of error compensating methods into metagenomic gene prediction tools as well as the development of suitable models specialized on the accurate detection of short gene fragments would be beneficial to improve metagenome annotation quality.

## Methods

### Read simulation

Sanger and 454 sequencing reads were simulating with MetaSim from the genomes of species given in Table 1 with 1-fold genome coverage as defined in [14,15].

Sanger reads were simulated with the error rates 0%, 0.0001% at the read start and 0.0002% at the read end, 0.001% to 0.002%, 0.01% to 0.02%, 0.1% to 0.2%, and 1% to 2%, and an average read length of 700 nt. Pyrosequencing reads were simulated with MetaSim from the same genomes, with an average read length of 450 nt, and with the error rates 0%, 0.22%, 0.49%, and 2.8% (1-fold genome coverage).

Further details on the simulation parameters of MetaSim are given in Additional file 1, section Supplementary methods.

### Benchmark data set

The FAMeS benchmark data sets simLC, simMC and simHC were retrieved from http://fames.jgi-psf.org/Retrieve_data.html in September 2008. For gene prediction accuracy assessment, the "genes that are included in the reference genomes" (further referred to as amino acid annotation file), and the "overlap of the genes with the sequencing reads" were also downloaded. The amino acid annotation file contains the full length amino acid sequences from genes in all genomes.

### Gene prediction

Genes in simulated reads and reads of the FAMeS data set were predicted with Genemark heuristic version 1.1, MetaGene and MetaGeneAnnotator as provided at http://metagene.cb.k.u-tokyo.ac.jp/metagene on February 1st 2009, respectively, and Orphelia as provided at http://orphelia.gobics.de/download.jsp on May 1st 2009. Concerning the two different models of Orphelia, we applied Net300 to all reads shorter or equal the length of 300 nt. Net700 was used for all remaining reads.

Genemark was run with the parameter -a to produce an amino acid sequence output. Amino acid sequences for the other tools were translated from nucleotide sequences that were excised from the simulated reads according to the predicted gene coordinates using BioPerl with the standard translation table [30].

### ESTScan training

For the application to metagenomic data, ESTScan 2.1 (available at http://sourceforge.net/projects/estscan) was trained with the annotated genes from the training genomes of MetaGene and Orphelia. Full coding regions were excised from the genomes with a flanking region of 50 nt. To directly obtain predicted amino acid sequences, ESTScan was applied to simulated and benchmark data with the option -t.

### Accuracy Assessment

Gene prediction accuracy was assessed through the alignment of amino acid sequences with BLAT. For simulated reads, the translation of annotated protein coding genes in the error free version of simulated reads were used as a reference. For the benchmark data set, full length amino acid sequences that completely or partically overlap with the reads were used as a reference.

True positives, false negatives and false positives are described in section 'Results', paragraph 'Accuracy on simulated genes'. Sensitivity and specificity are defined in equations 1 and 2:(1)

Sensitivity and specificity were combined into one measure by the harmonic mean (3):(3)

## Authors' contributions

KJH designed, conducted and evaluated all experiments. The author wrote, read and approved the final manuscript.

## Appendix: footnotes

^1 ^Error rates are given as 'error rate at the read start' to 'error rate at the read end'.

^2 ^Sensitivity (Sens.) expresses how many of the annotated genes were predicted.

^3 ^Specificity (Spec.) shows how many of the predicted genes were true.

^4 ^Low-complexity simulated data set with one dominating species and few reads from less abundant species.

^5 ^Medium-complexity simulated data set with several dominating species and reads form less abundant species.

^6 ^High-complexity simulated data set without dominating species.

## Supplementary Material

Additional file 1 **Supplementary Materials**. This pdf-file contains supplementary details to the section Methods, supplementary tables and supplementary figures. Click here for file
