# Supplementary material for: The effect of sequencing errors on metagenomic gene prediction
Source: BMC Genomics. 2009 Nov 12;10:520. doi: 10.1186/1471-2164-10-520 (PMC2781827; doi:10.1186/1471-2164-10-520)
Supplement: Additional file 1 — Supplementary Materials. This pdf-file contains supplementary details to the section Methods, supplementary tables and supplementary figures. [file 1471-2164-10-520-S1.pdf]

The effect of sequencing errors on metagenomic gene prediction – supplementary materials

Katharina J Hoff[1,2*]

[1]Department of Bioinformatics, Institute of Microbiology and Genetics, Georg-August-University Göttingen, Göttingen, Germany
[2]International Max Planck Research School for Molecular Biology, Georg-August-University Göttingen, Göttingen, Germany

Email: Katharina J Hoff*- katharina@gobics.de;

*Corresponding author

Supplementary tables

Table S1: **Amino acid prediction accuracy on simulated Sanger reads.**

	GeneMark		MetaGene		MGA		Orphelia		ESTScan	
Error rate[1]	Sens.[2]	Spec.[3]	Sens.[2]	Spec.[3]	Sens.[2]	Spec.[3]	Sens.[2]	Spec.[3]	Sens.[2]	Spec.[3]
0	96.1 ± 1.7	95.8 ± 2.5	95.2 ± 2.0	96.5 ± 1.3	96.1 ± 1.7	96.8 ± 1.3	95.0 ± 1.6	97.0 ± 1.3	82.4 ± 4.9	88.3 ± 2.0
$1 \times$ to 2×10^{-5}	96.1 ± 1.7	95.7 ± 2.7	95.5 ± 1.9	96.5 ± 1.3	96.0 ± 1.7	96.7 ± 1.4	95.1 ± 1.6	97.1 ± 1.3	82.6 ± 4.3	88.3 ± 2.1
$1 \times$ to 2×10^{-4}	95.5 ± 1.7	95.4 ± 2.6	94.9 ± 1.9	96.1 ± 1.4	95.5 ± 1.8	96.4 ± 1.4	94.2 ± 1.7	96.7 ± 1.4	82.4 ± 4.7	88.2 ± 2.0
$1 \times$ to 2×10^{-3}	89.8 ± 2.2	93.0 ± 2.9	89.8 ± 1.9	94.1 ± 1.8	90.4 ± 1.9	94.3 ± 1.7	87.2 ± 1.9	94.5 ± 2.2	80.8 ± 5.2	87.7 ± 2.1
$1 \times$ to 2×10^{-2}	53.5 ± 3.7	76.8 ± 7.4	54.2 ± 2.9	79.7 ± 5.0	55.7 ± 3.7	80.1 ± 5.0	44.1 ± 3.9	79.1 ± 5.7	61.1 ± 10.5	80.5 ± 2.7

Amino acid prediction accuracy of four metagenomic gene prediction tools GeneMark, MetaGene, MetaGeneAnnotator (MGA) and Orphelia, and of the EST processing tool ESTScan on simulated Sanger reads.

[1] Error rates are given as 'error rate at the read start' to (-) 'error rate at the read end'.
[2] Sensitivity (Sens.) expresses how many of the annotated genes were predicted.
[3] Specificity (Spec.) shows how many of the predcited genes were true.

Table S2: **Amino acid prediction accuracy on simulated 454 reads.**

	GeneMark		MetaGene		MGA		Orphelia		ESTScan	
Error rate	Sens.[1]	Spec.[2]	Sens.[1]	Spec.[2]	Sens.[1]	Spec.[2]	Sens.[1]	Spec.[2]	Sens.[1]	Spec.[2]
0	95.1 ± 2.2	95.1 ± 2.9	94.9 ± 2.1	95.9 ± 1.2	95.6 ± 1.8	96.3 ± 1.2	94.2 ± 1.9	96.3 ± 2.3	86.0 ± 5.8	91.7 ± 1.4
0.0022	77.1 ± 3.8	87.7 ± 4.7	79.2 ± 3.2	89.3 ± 2.9	80.1 ± 3.2	89.5 ± 2.7	73.3 ± 2.8	88.6 ± 5.3	79.4 ± 8.2	89.4 ± 1.6
0.0049	62.5 ± 3.9	81.2 ± 6.3	65.3 ± 3.0	83.5 ± 4.2	66.6 ± 3.4	83.8 ± 3.9	56.2 ± 4.4	82.1 ± 7.0	70.7 ± 10.8	86.5 ± 2.0
0.028	15.5 ± 1.9	50.3 ± 8.9	16.9 ± 1.7	52.9 ± 5.1	28.7 ± 2.6	53.3 ± 5.1	11.2 ± 4.3	45.4 ± 7.1	16.9 ± 8.4	62.5 ± 6.3

Amino acid accuracy of four metagenomic gene prediction tools GeneMark, MetaGene, MetaGeneAnnotator (MGA) and Orphelia, and of the EST processing tool ESTScan on simulated 454 reads.

[1]Sensitivity (Sens.) expresses how many of the annotated genes were predicted.
[2]Specificity (Spec.) shows how many of the predcited genes were true.

Supplementary figures

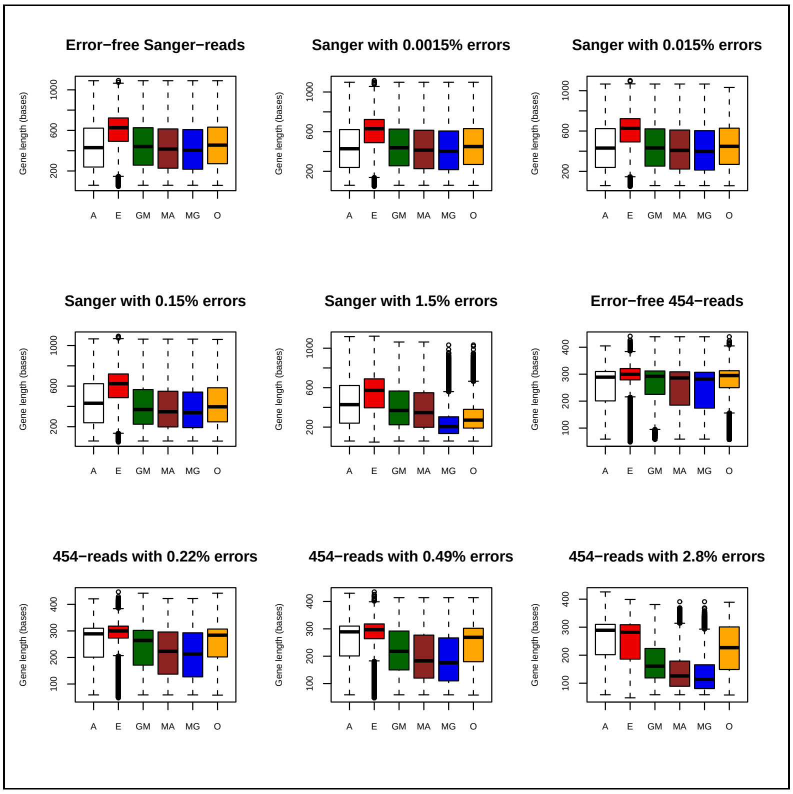

Figure S1: **G**ene lengths. Boxplots show the lengths of annotated genes on error free reads that correspond to reads with different error rates (A), and the lengths of genes predicted by GeneMark (GM), MetaGene (MG), MetaGeneAnnotator (MA), Orphelia (O), and ESTScan (E) on reads with the error rates specified in plot headers.

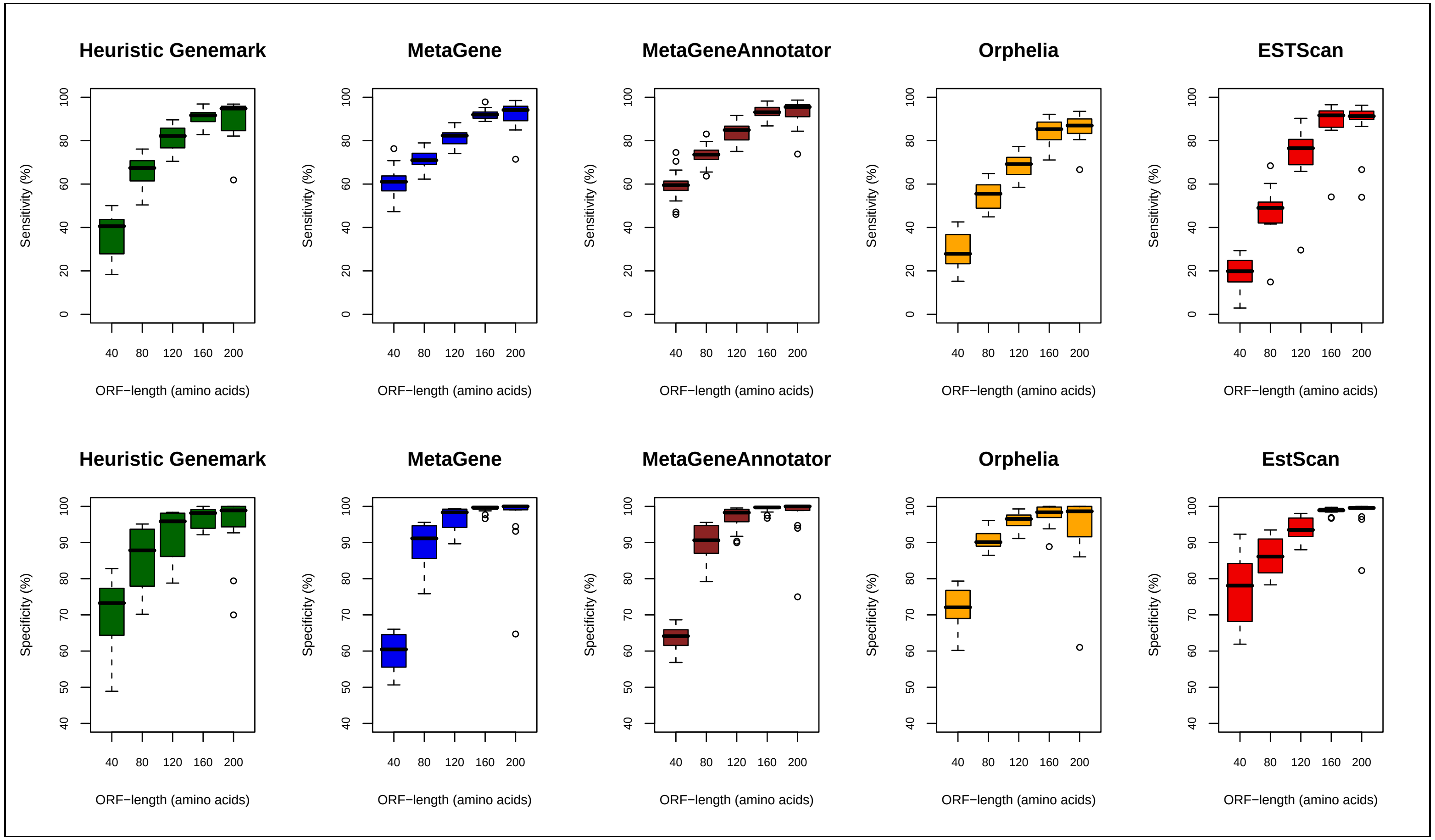

Figure S2: **G**ene prediction sensitivity and specificity for different ORF lengths.

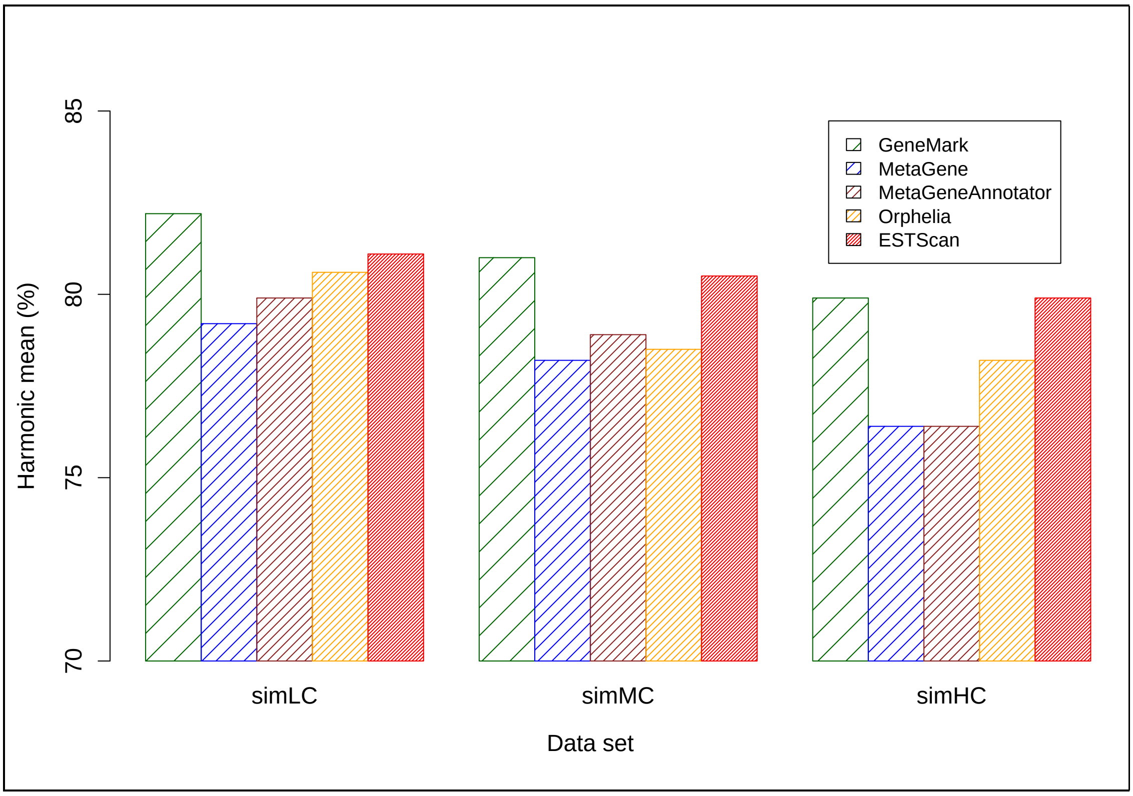

Figure S3: **A**verage gene prediction accuracy on FAMeS reads. Sensitivity and specificity were combined by the harmonic mean that is shown in this figure.

Supplementary methods

Read simulation with MetaSim

Sanger reads were sampled with the following general MetaSim simulation parameters (the error rates at beginning and end of read are given in the main manuscript):

```
Preset Name: Sanger
Number Of Reads / Mate Pairs=42417
Error Model=Sanger

Sanger Error Model Read Length Parameters=
Distribution: Normal
Mean: 700.0
2nd parameter: 100.0

Sanger Error Model Configuration=
Mate Pair Probability: 1.0
Proportion of Deletion Errors: 0.2
Proportion of Insertion Errors: 0.2
Proportion of Substitution Errors: 0.6000000000000001

Sanger Error Model DNA Clone Parameters=
Distribution: Normal
Mean: 5000.0
2nd parameter: 500.0

Combine All Files=false
Uniform Sequence Weights=false
Number Of Threads=1
Write FastA=true
Compress Output Files=false
```

Pyrosequencing reads were sampled with slightly different simulation parameters:

Preset Name: 454

Number Of Reads / Mate Pairs=98974

Error Model=454

454 Error Model Configuration=

Number Of Cycles: 176 (~450 Base Pairs)

Mate Pair Probability: 0.0

Mate Pair Read Length: 20

Remove Mate Pair Linker from Output: false

Scale Std. Deviation with Square Root of Mean: true

Generate Signal Trace: false

454 Error Model DNA Clone Parameters=

Distribution: Normal

Mean: 2000.0

2nd parameter: 200.0

Combine All Files=false

Uniform Sequence Weights=false

Number Of Threads=1

Write FastA=true

Compress Output Files=false

In addition, the lognormal distribution mean (LDM), the lognormal distribution standard deviation (LDSD), and the proportionality constant for standard deviation (PCSD) were individually set to achieve the simulation of different error rates (see table S):

Table S3: **Error rate specific simulation parameters for 454 reads**

Error rate	LDM	LDSD	PCSD
0	0.023	0.015	0.015
0.0022	0.08	0.09	0.09
0.0049	0.08	0.105	0.105
0.028	0.23	0.15	0.15

LDM, lognormal distribution mean; LDSD, lognormal distribution standard deviation; PCSD, proportionality constant for standard deviation.
